# Safety and tolerability of repeated doses of dihydroartemisinin-piperaquine for intermittent preventive treatment of malaria in pregnancy: a systematic review and an aggregated data meta-analysis of randomized controlled trials

**DOI:** 10.1186/s12936-023-04757-2

**Published:** 2023-10-21

**Authors:** Esther Nthenya Muthoka, Kedir Usmael, Saba Mehari Embaye, Abigiya Abebe, Tigist Mesfin, Dorothy Kazembe, Mediha Ahmedin, Stella Namuganza, Monica Kahabuka, Mary Gorret Atim, Tsegahun Manyazewal

**Affiliations:** 1 Centre for Innovative Drug Development and Therapeutic Trials for Africa (CDT-Africa), College of Health Sciences, Addis Ababa University, P.O. Box 9086, Addis Ababa, Ethiopia; 2 Tororo General Hospital, Tororo, Uganda; 3 Department of Medicine, College of Medicine and Health Sciences, Dire Dawa University, P.O. Box 1362, Dire Dawa, Ethiopia; 4 Department of Medicine, St. Paul’s Hospital Millennium Medical College, P.O Box 1271, Addis Ababa, Ethiopia; 5 St. Peter Specialised Hospital, Addis Ababa, Ethiopia; 6 Malawi Liverpool Wellcome Programme, Chichiri, P.O Box 30096, Blantyre 3, Malawi; 7 Addis Ababa Burn Emergency and Trauma Center, St. Paul’s Hospital Millennium Medical College, P.O Box 1271, Addis Ababa, Ethiopia; 8 Kibong’oto Infectious Disease Hospital, Mae Street, Lomakaa Road, P.O Box 12, Moshi-Kilimanjaro, Tanzania; 9 Kawempe National Referral Hospital, P.O Box 3253, Kampala, Uganda

**Keywords:** Malaria, Pregnancy, Intermittent preventive treatment, Dihydroartemisinin-piperaquine, Safety, Tolerability, Cardiotoxicity, Aggregated data meta-analysis, Randomised controlled trial

## Abstract

**Background:**

Malaria infection during pregnancy is an important cause of maternal and infant mortality and morbidity with the greatest effect being concentrated in sub-Saharan Africa. In areas of moderate to high malaria transmission, the World Health Organization (WHO) recommends the administration of intermittent preventive treatment of malaria in pregnancy (IPTp) using sulfadoxine-pyrimethamine (SP) to be given to all pregnant women at each scheduled antenatal care visit at monthly intervals. However, there is concern that increased resistance has compromised its effectiveness. This has led to a need for evaluation of alternatives to SP for IPTp with dihydroartemisinin-piperaquine (DP) emerging as a very promising candidate. Thus, this systematic review and aggregated data meta-analysis was conducted to establish the safety and tolerability of repeated doses with DP in IPTp.

**Methods:**

A systematic review and aggregated data meta-analysis of randomized controlled trials (RCTs) was performed by searching electronic databases of PubMed, Science Direct, ClinicalTrials.gov and Google Scholar. RCTs comparing IPTp DP versus recommended standard treatment for IPTp with these outcome measures were analyzed; change in QTc interval, serious adverse events (SAE), grade 3 or 4 adverse events possibly related to study drug and vomiting within 30 min after study drug administration. The search was performed up to 24th June 2023. Data was extracted from eligible studies and an aggregated data meta-analysis was carried out with data pooled as risk ratio (RR) with a 95% confidence interval (CI), using RevMan software (5.4). This study is registered with PROSPERO, CRD42022310041.

**Results:**

Six RCTs involving 7969 participants were included in this systematic review and aggregated data meta-analysis. The pooled analysis showed that DP was associated with a change from baseline of the QTc interval although this change was not associated with cardiotoxicity. There was no statistically significant difference in the risk of occurrence of SAEs among participants in both treatment groups (RR = 0.80, 95% CI [0.52–1.24], P = 0.32). However, significant difference was observed in grade 3 or 4 AEs possibly related to study drug where analysis showed that subjects on IPT DP were statistically significantly more likely to experience an AE possibly related to study drug than subjects on IPT SP (RR = 6.65, 95% CI [1.18–37.54], P = 0.03) and in vomiting within 30 min after study drug administration where analysis showed that the risk of vomiting is statistically significantly higher in subjects receiving IPT DP than in subjects receiving IPT SP (RR = 1.77, 95% CI [1.02–3.07], P = 0.04).

**Conclusion:**

DP was associated with a higher risk of grade 3 or 4 AEs possibly related to study drug and a higher risk of vomiting within 30 min after study drug administration. However, these were experienced in a very small percentage of women and did not affect adherence to study drugs. DP was also better tolerated in these studies as compared to most alternatives that have been proposed to replace SP which have proved to be too poorly tolerated in IPTp use.

## Background

Due to changes in women’s immune systems during pregnancy and the presence of the placenta for which parasites have a high binding affinity, pregnant women are prone to malaria infection [1]. Malaria infection during pregnancy has a devastating effect on the health of mothers and their babies, and is an important cause of maternal and infant mortality and morbidity [2]. It is associated with maternal anaemia, infant low birth weight, fetal loss, premature delivery and intrauterine growth retardation [3, 4]. In particular, malaria is a problem for women in their first and second pregnancies and for women who are HIV-positive. Pregnant women have higher prevalence and densities of parasitaemia than other women from the same population [2, 5]. Adverse effects vary by transmission level. In areas of high transmission, because of developed immunity severe disease may not occur; however, during pregnancy, parasites specifically target the placenta leading in an increased risk and the level of immunity may also be diminished during pregnancy. In areas of low transmission, women have not yet developed immunity to malaria and infection is more likely to lead to severe malaria disease [1].

Most of the effect of malaria in pregnancy is concentrated in sub-Saharan Africa and is mainly caused by *Plasmodium falciparum* infection. In 2019, in 33 moderate to high transmission countries in the WHO Africa Region, there were an estimated 33 million pregnancies, of which 35% (12 million) were exposed to malaria infection during pregnancy. Of all the World Health Organization (WHO) sub-regions, Central Africa had the highest prevalence of exposure to malaria during pregnancy (40%), closely followed by West Africa (39%), while prevalence was 24% in East and Southern Africa. It is estimated that malaria infection in these 33 countries resulted in 822,000 children with low birth weight [6].

### Malaria prevention in pregnancy

The WHO recommends a package of interventions for controlling malaria and its effects during pregnancy in areas of moderate to high transmission of *P. falciparum,* which include promotion and use of insecticide treated nets (ITNs), the administration of IPTp using SP, indoor residual spraying and appropriate case management through prompt and effective treatment of malaria in pregnant women [7]. By 2019, 68% of households in sub-Saharan Africa had at least one ITN. The percentage of pregnant women sleeping under an ITN was 52%. To date, 33 African countries have adapted IPTp with SP to reduce the burden of malaria during pregnancy. The percentage of IPTp use by dose computed using data from the 33 African countries in 2019 was; IPTp1 about 62%, IPTp2 49% and IPTp3 34% [6]. In areas of moderate-to-high malaria transmission in Africa, the WHO encourages IPTp with SP to be given to all pregnant women at each scheduled antenatal care visit, starting as early as possible in the second trimester until the time of delivery, provided that the doses of SP are given at least one month apart with an objective to ensure that at least three doses are received [8].

At a WHO evidence review, a meta-analysis of seven trials evaluating IPTp-SP showed that three or more doses of IPTp-SP were associated with higher mean birth weight and fewer low birth weight births than two doses of IPTp-SP. The three and above dose group was also found to have less placental Malaria. IPTp-SP should ideally be administered as directly observed therapy (DOT) of three tablets of SP each tablet containing 500 mg/25 mg with or without food [9]. Despite historical evidence for benefits of IPTp with SP, there is concern that increased resistance has compromised its effectiveness. In much of Eastern and Southern Africa, 90% of parasites harbour five mutations [10]. SP resistance is linked with dihydrofolate reductase (*dhfr*) in the folate biosynthetic pathway and substitutions of amino acids in the enzyme dihydropteroate synthase (*dhps*) [11]. Resistance to SP is caused by point mutations in the *dhfr* and *dhps* genes of *P. falciparum* at codons 51,59,108, and 164 within *pfdhfr* and codons 437,540 and 581 within *pfdhps* [12]. The combination of a triple *dhfr* mutant with a double *dhps* mutant is a useful predictor of clinical SP treatment failure and results in limited efficacy of SP-IPT. The more mutations accumulate in these genes, the greater the amount of resistance conferred to the parasite [13]. The WHO recommendation is that intermittent preventive treatment of malaria in infants with sulfadoxine-pyrimethamine (SP-IPTi) should not be implemented when the prevalence of *dhps* K540E exceeds 50% [14]. A recent meta-analysis demonstrated that IPTp efficacy was reduced when the prevalence of A581G exceeds 10% in Africa [15]. Resistance to SP has become widespread, especially in Eastern Africa and Southern Africa. Recent studies have also suggested that the effectiveness of this drug as IPTp maybe compromised [16–18]. This situation may suggest discontinuation of IPTp -SP. In some parts of East Africa, other findings reported that IPT-SP as IPTp has failed in the quintuple mutant N511/C59R/S108N + A437G/K540E acquired *pfdhps* A581G [19, 20].

Thus, there is a need for evaluation of alternatives to SP for IPTp with dihydroartemisinin-piperaquine (DP) emerging as a favourite candidate. Dihydroartemisinin-piperaquine is an attractive alternative to SP for IPTp because it is highly efficacious in eliminating malaria parasites and the long half-life of piperaquine provides at least four weeks of post-treatment prophylaxis [21, 22]. Recent studies from East Africa have shown that IPTp with DP was more effective than SP at reducing the risk of placental malaria at delivery. Monthly DP was associated with a lower incidence of symptomatic malaria, a lower prevalence of parasitaemia during pregnancy, less moderate-to-high-grade placental pigment deposition, and a lower risk of any adverse birth outcome [23, 24]. Two systematic reviews comparing DP versus SP for IPTp also concluded that IPTp with DP was a promising alternative in a setting with high SP resistance. However, more data was required to identify the risk of adverse events [25, 26]. In 2015, a WHO Malaria Policy Advisory Committee concluded that IPTp with DP requires further study [9].

### Concern about the use of dihydroartemisinin-piperaquine

DP is an effective artemisinin-based combination antimalarial therapy. The long-term elimination half-life of piperaquine (20–30 days) provides a long post- treatment prophylactic effect that makes it a candidate for IPT. However, piperaquine has been associated with a dose dependent prolongation of cardiac ventricular repolarization duration and QT interval, leading to concerns about its potential to cause lethal ventricular tachyarrhythmias. Extreme prolongation of the QT interval can lead to *torsades de pointes* (TdP) a polymorphic ventricular tachycardia that can degenerate in some cases into ventricular fibrillation and lead to sudden cardiac death. Prolongation of the QT/QTc interval is a sensitive but not specific marker of an increased risk of TdP. It is at present the best available surrogate indicator for TdP risk. A number of correction formulae accounting for the inverse relationship between QT interval and heart rate are used to routinely adjust measured QT interval for heart rate. This corrected QT value is referred to as QTc. No agreement has been reached yet concerning what the upper limit values for absolute QT/QTc interval and changes from baseline should be. Lower limits are likely to increase the false- positive rate while high limits likely increase the risk of failing to detect a signal for concern. The threshold of major concern in clinical trials during therapy is a QTc prolongation > 500 ms. One way to approach this uncertainty is to conduct multiple analyses using different limits, including absolute QTc interval prolongation (QTc interval > 450 ms, QTc interval > 480 ms, QTc interval > 500 ms) and change from baseline in QTc interval (QTc interval increases from baseline > 30 ms, QTc interval increases from baseline > 60 ms).

In malaria-endemic regions, there is limited access to ECG monitoring for arrhythmia detection thus understanding the frequency of drug-related sudden death is key to assessing the risk of DP cardiotoxicity [27]. Limited data exists on whether the risk of QT prolongation is increased with repeated dosing. The slow elimination of piperaquine also poses the question of whether this risk is not increased when repeated doses are given especially when given monthly. Two systematic reviews and meta-analysis conducted on the safety of DP only included two RCTs with data on IPT in pregnant women [28, 29]. This shows that data on the safety of DP use as IPTp is inadequate.

Hence, this study aimed to assess the safety and tolerability of repeated doses of DP for intermittent preventive treatment of malaria in pregnancy through a systematic review and an aggregated data meta-analysis of randomized controlled trials.

## Methods

The protocol for this systematic review and meta-analysis has been registered at the International Prospective Register of Systematic Reviews (PROSPERO) database, ID: CRD42022310041. The Preferred Reporting Items for Systematic Reviews and Meta-Analyses( PRISMA) guidelines [30] was followed to choose studies to be included in this review.

### Inclusion criteria

Trials were considered eligible for this systematic review using the following PICOS format:

#### Population

Pregnant women and adolescents in their second and third trimester.

#### Intervention

Intermittent Preventive Treatment with Dihydroartemisinin-Piperaquine.

#### Comparator

Recommended standard treatment for IPTp i.e. IPTp with SP or IPTp with Trimethoprim/Sulfamethoxazole (TMP/SMX).

### Outcomes

Provided information on one or more of these outcome measures; change in QTc interval, serious adverse events, grade 3 or 4 adverse events possibly related to study drugs and vomiting within 30 min after study drug administration.

### Study type

Randomized controlled trials published in English. Studies were excluded if they did not have results, if they were post hoc analysis from previous controlled studies and if they were unpublished.

### Systematic search of literature

Relevant studies were identified from electronic searches of these databases; PubMed, Science Direct, Clinicaltrials.gov and Google Scholar. Published studies in the English language were searched up to 24th June 2023 without restriction in the year of publication. Key search terms used in different combinations were; intermittent preventive treatment, malaria, pregnancy, Dihydroartemisinin-Piperaquine, cardiotoxicity, cardiac safety, QT interval prolongation, randomized controlled trial and clinical trials.

### Study selection

This systematic review and meta-analysis was conducted according to the PRISMA guidelines and the Cochrane Handbook for the Systematic Reviews of Interventions [30, 31]. The titles of all searched studies were read and those that clearly did not conform to the inclusion criteria and duplicated studies were excluded. Then, abstracts and full texts of the remaining studies were reviewed by two independent review authors to identify those that satisfied the inclusion criteria.

### Data extraction

Two review authors independently extracted the following data from each included study:

General Information including; 1. First author, year of publication, number of participants randomized, intervention and comparator. 2. Safety and tolerability data including change in QT interval, incidence of serious adverse events (SAEs), incidence of grade 3 and 4 adverse events possibly related to study drug and prevalence of vomiting within 30 min after study drug administration.

### Assessment of risk of bias

The Cochrane risk of bias tool was used to assess the risk of bias for included studies. Risk of bias was judged as low, unclear or high based on these domains: Sequence generation, Allocation concealment, blinding of participants, Personnel and outcome assessors, Incomplete outcome data, Selective outcome reporting and other sources of bias.

### Measures of effect

The measure of effect used was risk ratio for SAEs, grade 3 or 4 AEs possibly related to study drug and vomiting after study drug administration. For change in QTc interval, mean change was reported.

### Assessment of heterogeneity

Heterogeneity among trials was assessed by visually inspecting forest plots to assess for overlapping CIs. The amount of overall heterogeneity between studies was measured using the I^2^ statistic. It was categorized as low if I^2^ was below 25%, moderate if I^2^ was below 50% and high if I^2^ was above75% following the Cochrane Handbook for Systematic Reviews of Interventions Version 6.0, chapter 10: Analyzing data and undertaking meta-analyses [31].

### Statistical analysis

All statistical analysis were performed using the Review Manager 5.4. QTc interval change was reported using mean change. Pooled relative risks were generated for SAEs, grade 3 or 4 AEs possibly related to study drugs and vomiting after drug administration with 95% CI using random- effect model meta-analysis. Mantel–Haenszel random-effect meta-analysis was performed in consideration of heterogeneity between studies.

## Results

### Study selection

**Fig. 1:**
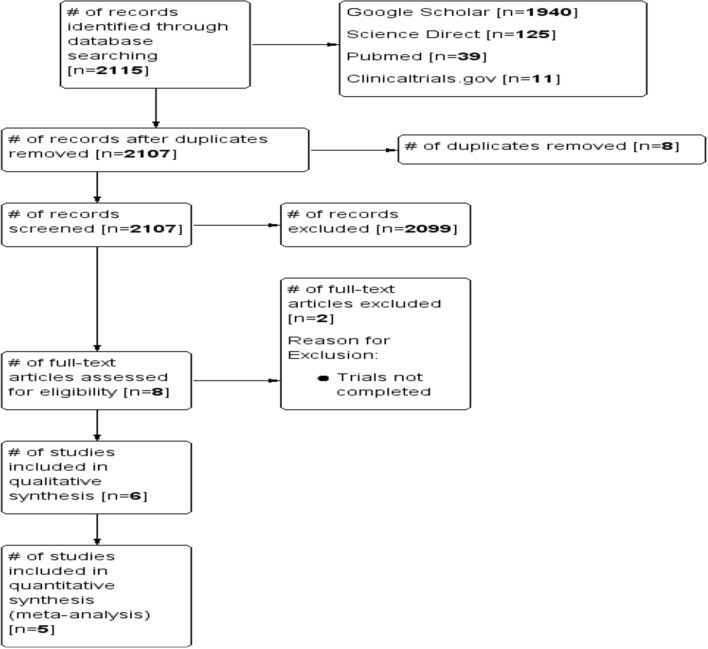
PRISMA Flow Diagram of search results

As shown in Fig. 1, a total of 2115 citations were searched through electronic database search, of which 8 were duplicates. The remaining 2107 were screened, out of which 8 full text articles were assessed for eligibility. Among the 8 full text articles, 6 RCTs [10, 23, 24, 32–34] fulfilled the inclusion criteria and none of the exclusion criteria. The reasons for excluding 2 potential full text articles got from Clinical Trials.gov was that results were not published, (NCT02909712, NCT03009526).

### Characteristics of included studies

Table 1 summarizes the characteristics of included studies. Among the 6 included studies, one study [32] used daily Trimethoprim-Sulfamethoxazole (TMP-SMX) as the comparator as compared to monthly SP used by the rest of the studies and monthly DP + daily TMP/SMX as intervention compared to monthly DP used in the other studies. It also included HIV-infected pregnant women as the study population. Another study [24] used 3 doses of SP as comparator as compared to monthly SP used in the other studies. Three studies [10, 24, 32] had results for the outcome change in QTc interval, five studies [10, 23, 24, 32, 34] reported on the outcome SAEs, two [10, 24] reported on grade 3 and 4 AEs possibly related to study drugs and five [10, 24, 32–34] on vomiting within 30 min after study drug administration. Finally one study [34] had a third arm that had DP + Azithromycin as intervention.

**Table 1:** Characteristics of included studies

Author, publication year	Setting	Sample size (n)	Intervention (n of patients)	Comparator (n of patients)	Important patient outcomes
Desai et al. 2015 [23]	Kenya	n = 1546	IPT DP (n = 516)	IPT SP (n = 515) IST DP (n = 515)	Incidence of SAEs
Mlugu et al. 2021 [33]	Tanzania	n = 956	IPT DP (n = 478)	IPT SP (n = 478)	Prevalence of vomiting within 30 min after study drug administration
Kajubi et al. 2019 [10]	Uganda	n = 782	IPT DP (n = 391)	IPT SP (n = 391)	Mean change in QTc interval Incidence of SAEs Incidence of grade 3 or 4 AEs possibly related to study drug Prevalence of vomiting within30 minutes after study drug administration
Kakuru et al. 2016 [24]	Uganda	N = 300	IPT DP 3 doses (n = 94) IPT DP Monthly (n = 100)	IPT SP 3 doses (n = 106)	Median change in QTc interval Incidence of SAEs Incidence of grade 3 or 4 AEs possibly related to study drug Prevalence of vomiting within 30 min after study drug administration
Natureeba et al. 2017 [32]	Uganda	n-200	IPT DP Monthly + TMP/SMX Daily (n = 100)	TMP/SMX Daily(n = 100)	Mean change in QTc interval Incidence of SAEs Prevalence of vomiting within 30 min after study drug administration
Madanitsa et al. 2023 [34]	Kenya, Malawi, Tanzania	N = 4680	IPT DP (n = 1561) IPT DP + Azithromycin (1558)	IPT SP (n = 1561)	Incidence of SAEs Vomiting within 30 min after study drug administration

*IPT* intermittent preventive treatment, *SP* sulfadoxine−pyrimethamine, *DP* dihydroartemisinin−piperaquine, *TMP* trimethoprim, *SMX* sulfamethoxazole, *n* number, *SAE* serious adverse events

### Risk of bias in included studies

**Fig. 2:**
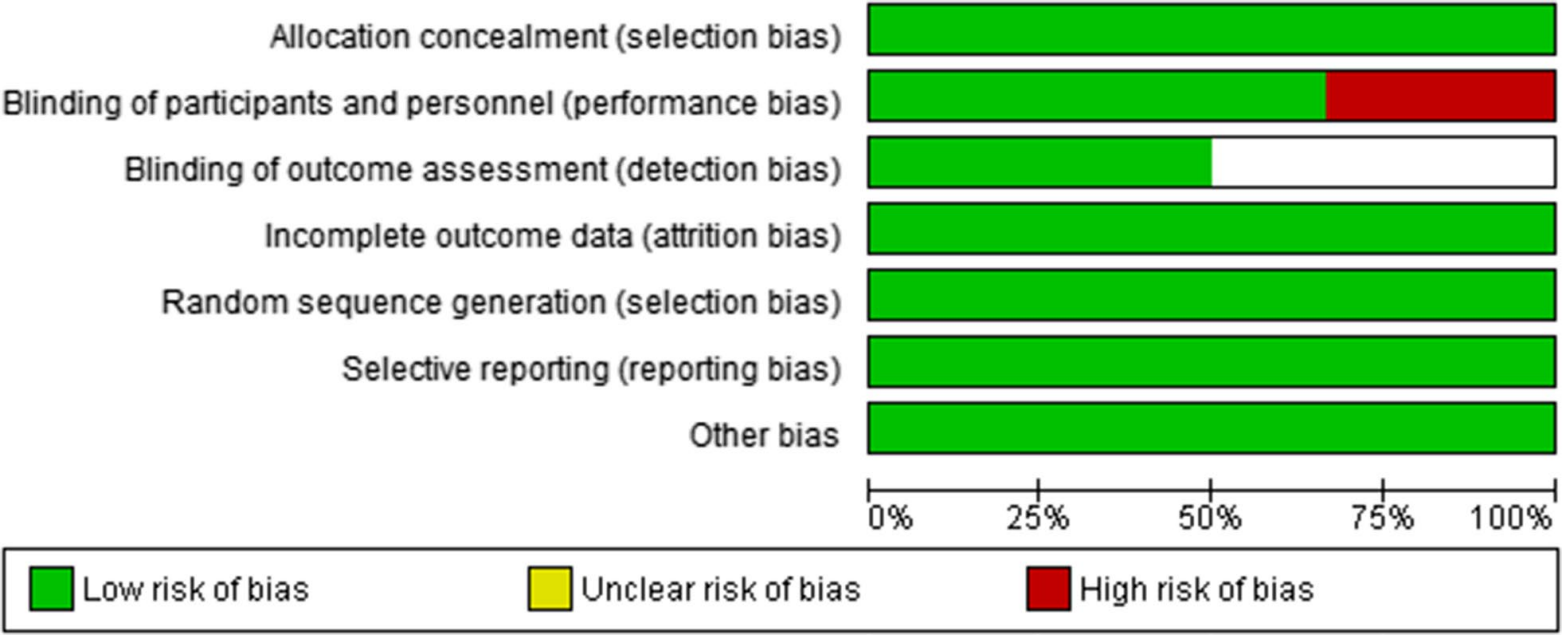
Risk of bias graph: review authors’ judgements about each risk of bias item presented as percentages across all included studies

**Fig. 3:**
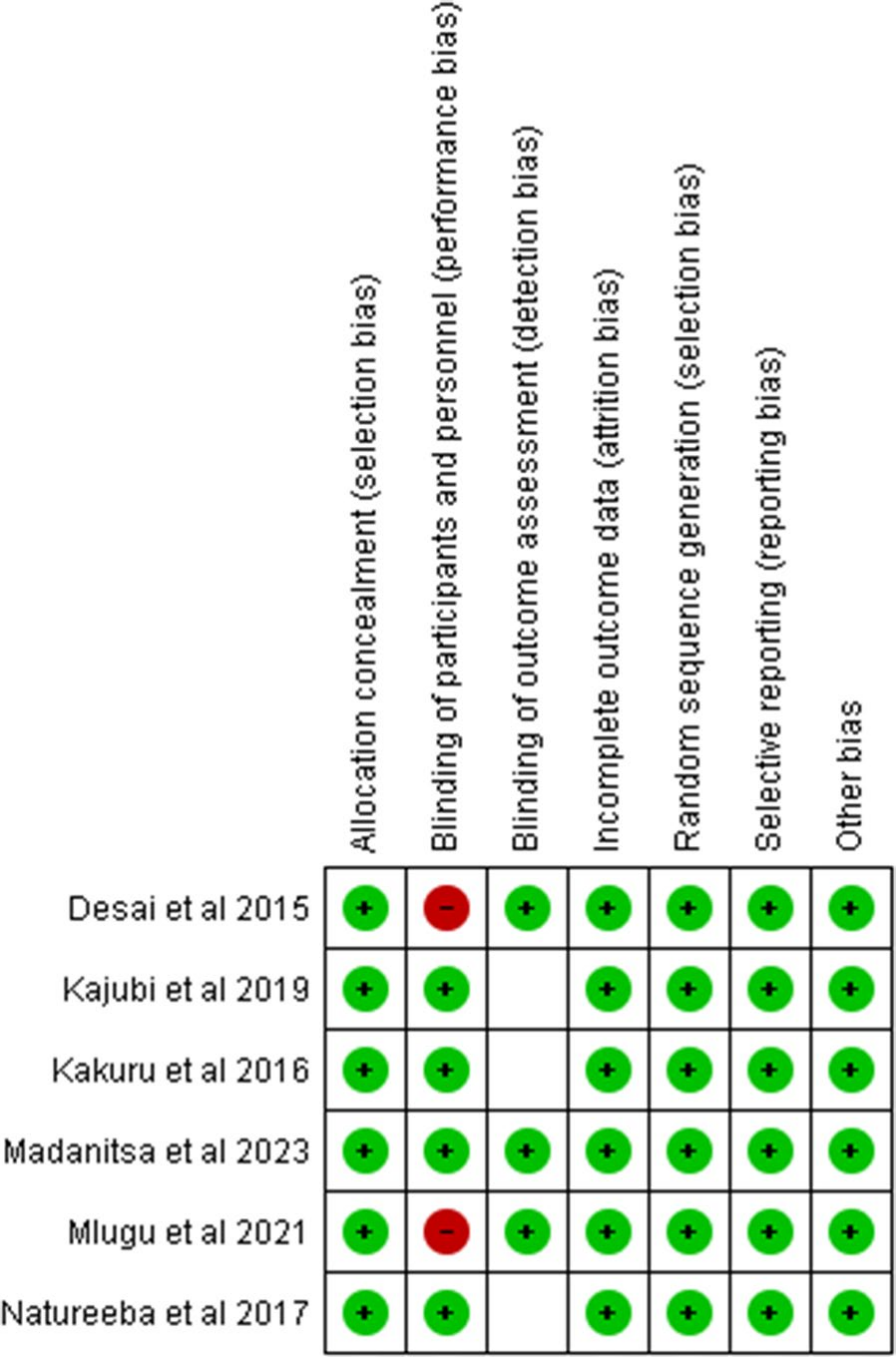
Risk of bias summary: review authors’ judgements about each risk of bias item for each included study. (Green cells = ‘low risk’; Red cells = ‘high risk’)

As illustrated in Figs. 2 and 3, the risk of bias in included studies was found to be ‘low risk of bias’ when these studies were subjected to the Cochrane Risk of bias assessment tool.

### Safety and tolerability assessments

#### Change in QTc interval.

Three RCTs [10, 24, 32] assessed mean/median change in QTc interval in a total of 879 participants. A higher mean change in QTc interval was observed in subjects on IPT DP as compared to those on IPT SP or TMP/SMX. However, none of the studies reported a mean change greater than 60mSec. (Table 2).

**Table 2:** Change in QTc interval

Author, publication year	Sample size (n)	SP	DP
Kajubi et al. 2019 [10]	782	0mSec	13mSec
Kakuru et al. 2016 [24]	42	5mSec	30mSec
		**TMP-SMX**	**TMP-SMX/DP**
Natureeba et al. 2017 [32]	55	0mSec	15mSec

*SP* sulfadoxine−pyrimethamine, *DP* dihydroartemisinin−piperaquine, *TMP* trimethoprim, *SMX* sulfamethoxazole, *n* number

#### Serious adverse events

**Fig. 4:**
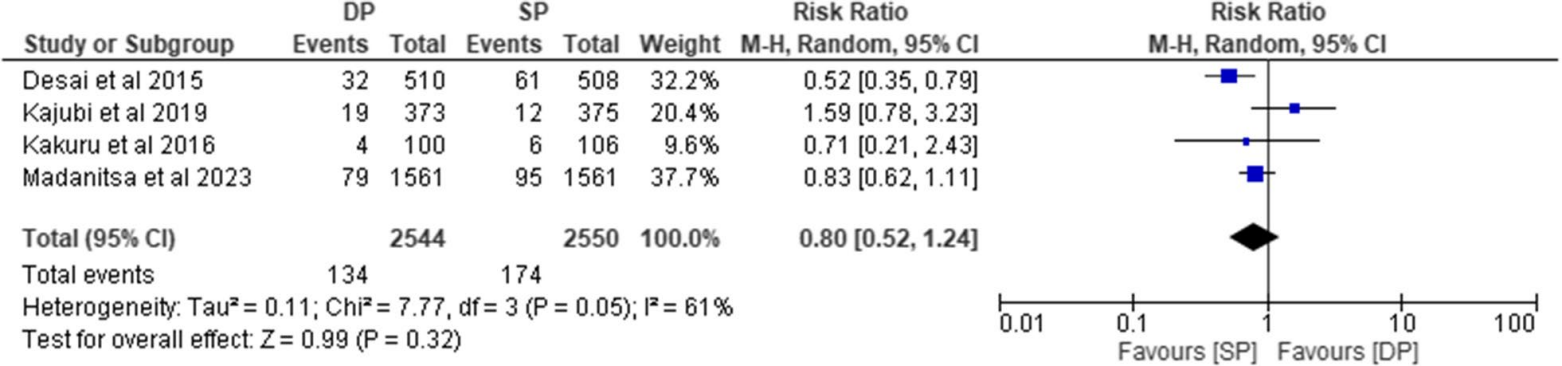
*Comparison of serious adverse events between DP and SP.* *RR* Risk Ratio, CI = Confidence Interval, *DP* Dihydroartemisinin-Piperaquine, *SP* Sulfadoxine- pyrimethamine

Five trials [10, 23, 24, 32, 34], assessed this outcome where pooled risk ratios of four of the studies [10, 23, 24, 34] showed that there was no significant difference in the occurrence of serious adverse events between subjects on IPT DP versus subjects on IPT SP as evidenced by a P value of 0.32 (RR = 0.80, 95% CI [0.52–1.24], P = 0.32). There was moderate heterogeneity between studies as shown by (I^2^ = 61%; P = 0.05) (Fig. 4). Also, the trial comparing TMP-SMX + DP versus TMP-SMX did not show significant difference in occurrence of SAEs between the two groups.

#### Grade 3 or 4 AEs possibly related to study drug

**Fig. 5:**
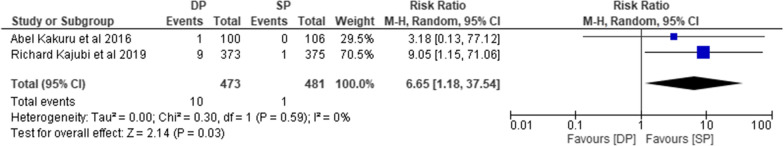
*Comparison of grade 3 or 4 adverse events possibly related to study drug between DP and SP.* *RR* Risk Ratio, CI Confidence Interval, *DP* mDihydroartemisinin-Piperaquine, *SP* Sulfadoxine- pyrimethamine

Two trials, [10, 24] assessed for this outcome where the pooled analysis of the risk of a grade 3 or 4 AE possibly related to study drug occurring showed that subjects on IPT DP were statistically significantly more likely to experience an AE possibly related to study drug than subjects on IPT SP. (RR = 6.65, 95% CI [1.18–37.54], P = 0.03). There was no heterogeneity between studies (I^2^ = 0%; P = 0.59) (Fig. 5).

#### Vomiting within 30 min after study drug administration.

**Fig. 6:**
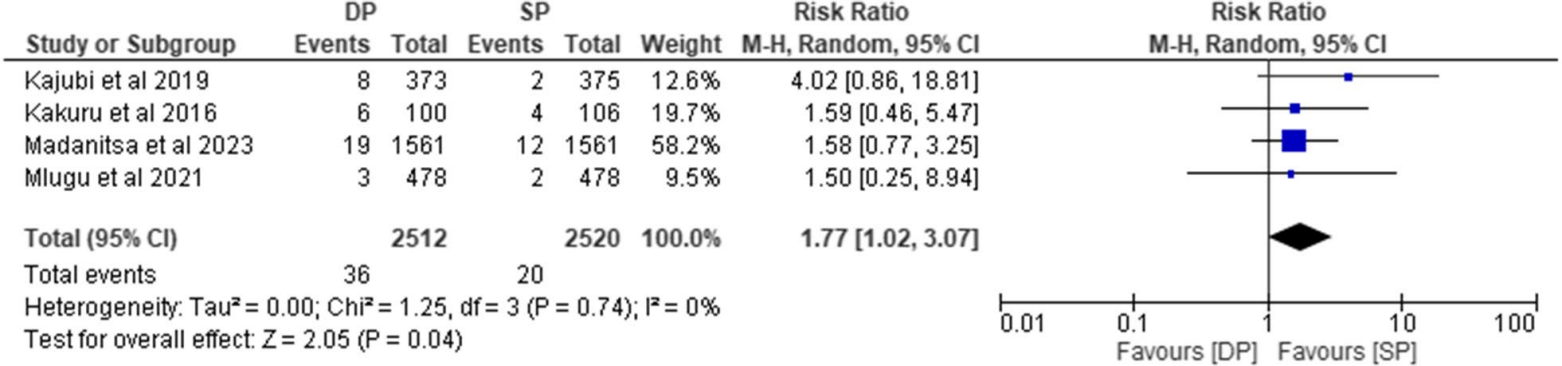
*Comparison of vomiting after study drug administration between DP and SP.* *RR* Risk Ratio, *CI* Confidence Interval, *DP* Dihydroartemisinin-Piperaquine, *SP* Sulfadoxine- pyrimethamine

This outcome was assessed by five studies [10, 24, 32–34]. The pooled analysis of four of the studies [10, 24, 33, 34] showed that the risk of vomiting within 30 min after study drug administration is statistically significantly higher in subjects receiving IPT DP than in subjects receiving IPT SP (RR = 1.77, 95% CI [1.02–3.07], P = 0.04). There was no heterogeneity between studies (I^2^ = 0%;P = 0.74) (Fig. 6). Also the trial comparing TMP-SMX versus TMP-SMX + DP reported a higher number of participants on the DP arm experiencing vomiting after study administration as compared to the TMP-SMX arm.

## Discussion

Dihydroartemisinin piperaquine has been shown to cause prolongation of QTc interval leading to a concern of the potential of repeated doses to cause life threatening cardiotoxicity. Despite two systematic reviews assessing the safety and tolerability of repeated doses of DP concluding that the risk of cardiotoxicity was not significant, very limited data on pregnant women was included [28, 29]. This systematic review and aggregated data meta-analysis of published evidence is a more comprehensive attempt to assess the safety and tolerability of repeated doses of DP in pregnant women.

This study showed that indeed DP is associated with changes in QTc interval as is consistent with available literature. Three studies with data on QTc interval change on 879 participants showed a change in QTc interval from baseline. However, none of the studies reported change above 30mSec which is the lower threshold for change from baseline and also none reported QTc intervals above 500mSec which is the upper ‘at risk’ threshold for QTc interval prolongation. None of these changes were associated with any clinically significant change echoing findings by Ahmed *et al.* and Hughes *et al.* who also established that QTc prolongation decreased with each repeat dose [35, 36]. The findings are consistent with the recommendation of a WHO Malaria policy advisory committee meeting [9] which concluded that DP has a low risk of cardiotoxicity that is similar to that of other antimalarial drugs including quinine, chloroquine and amodiaquine. Also, according to Borsini *et al.* [37] in their in vitro study, despite significant hERG blockage piperaquine does not appear to induce torsadogenic effects in vitro. With regard to severe adverse events this study did not find any significant differences in the risk of occurrence of SAEs among the different treatment groups echoing what individual studies also found. DP was associated with a higher risk of grade 3 or 4 adverse events possibly related to study drugs in this study. Both the trials analysed for this outcome were placebo controlled. DP was also associated with a slightly significant higher risk of vomiting after drug administration. However, despite vomiting being experienced more in the groups taking DP, it was experienced by less than 3% of women and did not affect adherence and dropouts in the individual studies. DP was better tolerated in these studies as compared to most alternatives that have been proposed to replace SP which have proved to be too poorly tolerated in IPTp use. These include amodiaquine alone or in combination with SP [38], mefloquine monotherapy [39, 40] and a fixed dose combination of chloroquine and azithromycin [41]. Individual trials also suggested no statistically significant differences in the occurrence of adverse events and vomiting.

### Limitations

The most important limitation of this systematic review and meta-analysis was that data was analyzed from a few studies. The other limitation was that data for QTc prolongation from the studies analyzed was obtained from few participants and may not be truly representative of the outcome.

Also, one trial used a different dose for IPT SP from the rest of trials and could have also accounted for heterogeneity. Finally, it is possible that restricting to English language excluded relevant studies published in other languages.

## Conclusion

Despite non-significant difference in serious adverse events among the different treatment groups, DP was associated with a higher risk of grade 3 or 4 adverse events possibly related to drug and a higher risk of vomiting within 30 min after study drug administration. However, DP was better tolerated as compared to most alternatives to replace SP and adherence to DP was also not affected.

However, despite QTc prolongation with DP seemingly not being a limiting factor for repeat dosing, life-threatening QTc prolongation such as Torsades de Pointes in patients with pre-existing long QT intervals is rare and thus requires a much larger sample size to provide reasonable reassurance. Thus more studies on this will be required to achieve reassurance.

## Data Availability

All data generated or analyzed during this study are included in this published article.

## Abbreviations

ACT: Artemisinin-based combination therapy
AEs: Adverse events
CDC: Centers for disease control and prevention
CI: Confidence interval
pfdhfr: Plasmodium falciparum dihydrofolate reductase
pfdhps: Plasmodium falciparum dihydropteroate synthase
dhfr: Dihydrofolate reductase
dhps: Dihydropteroate synthase
DNA: Deoxyribonucleic acid
DOT: Directly observed therapy
DP: Dihydroartemisinin-piperaquine
DRC: Democratic Republic of Congo
ECG: Electrocardiogram
IPT: Intermittent preventive treatment
IPTi: Intermittent preventive treatment of malaria for infants
IPTp: Intermittent preventive treatment of malaria in pregnancy
ITN(s): Insecticide-treated bed nets
PABA: Para-amino benzoic acid
PLWHIV: People living with human immuno-deficiency virus
PRISMA: Preferred reporting items for systematic reviews and meta-analysis
RR: Risk ratio
SAEs: Serious adverse events
SP: Sulfadoxine-pyrimethamine
TdP: Torsades de Pointes
TMP-SMX: Trimethoprim-sulfamethoxazole
WHO: World Health Organization
